# Supplementary material for: Heat adaptation of phage T7 under an extended genetic code
Source: Virus Evol. 2021 Dec 1;7(2):veab100. doi: 10.1093/ve/veab100 (PMC8923235; doi:10.1093/ve/veab100)
Supplement: veab100_Supp [file veab100_supp.zip › Sequencing_Analysis.zip!/Sequencing_Analysis/A1/evidence/JC_SIDE_2_62.html]

BRESEQ :: Evidence


| New junction evidence | | | | | | | | | | | |
| --- | --- | --- | --- | --- | --- | --- | --- | --- | --- | --- | --- |
|  | | seq id | position | reads (cov) | reads (cov) | score | skew | freq | annotation | gene | product |
| \* | ? | NC\_001604.1 | = 13586 | 3 (0.000) | 2345 (0.770) | 347/592 | 0.3 | 93.9% | intergenic (–/–) | *–/–* | –/– |
|  | ? | NC\_001604.1 | 14356 = | 301 (0.100) |  |  |  |  | intergenic (–/–) | *–/–* | –/– |

Only 100 of 3225 total aligned reads displayed.  

`GAAGAAACCAGAAGTTACCAAGCTGAGTCATAAGGTGTTCGATAAGGACACTATGACCCACATCAAGGCTGGTGATTGGGTTAAGGTTGACATGGGAGTTGTTGGTGGATACGGCTACGTCCGCTCAGTTAGTGGCAAATATGCACAAGTGTCATACATCACAGGTGTTACTCCACGCGGTGCAATCGTTGCCGATAAGACCAACATGATTCACACAGGTTTCTTGACAGTTGTTTCATATGAAGAGATTGTTAAGTCACGATAATC‑AATAGGAGAAATCAATATGATCGTTTCTGACATCGAAGCTAACGCCCTCTTAGAGAGCGTCACTAAGTTCCACTGCGGGGTTATCTACGACTACTCCACCGCTGAGTACGTAAGCTACCGTCCGAGTGACTTCGGTGCGTATCTGGATGCGCTGGAAGCCGAGGTTGCACGAGGCGGTCTTATTGTGTTCCACAACGGTCACAAGTATGACGTTCCTGCATTGACCAAACTGGCAAAGTTGCAATTGAACCGAGAGTTCCACCTTCCTCGTGAGAACTGTATTGACACCCTTGTGTTGTCACGTTTGATTCATTCCAACC  >  NC_001604.1/14070‑14656`  

                                                                                                                                                                                                                                                                                               `|`                                                                                                                                                                                                                                                                                                              
`aggtttcgctggagagtcccagtctaatacgactcactaaaggagacacaccatgttcaaactgattaagaagttaggccaactgctggttcgtatgtacaacgtggaagccaagcgactgaacgatgaggctcgtaaagaggccacacagtcacgcgctctggcgattcgctccaacgaactggctgacagtgcatccactaaagttaccgaggctgcccgtgtggcaaaccaagctcaacagctttccaaattctttgagtaatcaaacaggagaaataaatatgATCGTTTCCAACAt                                                                                                                                                                                                                                                                                                 <  1:395010‑M2/14‑1 (MQ=255)`  
  `gtttcgctggagagtcctattctaatacgactcactaaaggagacacaccatgttcaaactgattaagaagttaggccaactgctggttcgtatgtacaacgtggaagccaagcgactgaacgatgaggctcgtaaagaggccacacagtcacgcgctctggcgattcgctccaacgaactggctgacagtgcatccactaaagttaccgaggctgcccgtgttgcaaaccaagctcaacagctttccaaattctttgagtactcaaacaggagaaaccattatgatcgctgttccttaaa                                                                                                                                                                                                                                                                                               >  1:224812‑M2/286‑289 (MQ=255)`  
     `tcgctggagagtcccattctaatacgactcactaaaggagacacaccatgttcaaactgattaagaagttaggccaactgctggttcgtatgtacaacgtggaagccaagcgactgaacgatgaggctcgtaaagaggctacacagtcacgcgctctggcgattcgctccaacgaactggctgacagtgcatccactaaagttaccgaggctgcccgtgtggcaaaccaagctcaacagctttccaaattctttgagtaatcaaacagtagaaaccattatgATCGTTTCTGACATCGAAg                                                                                                                                                                                                                                                                                            >  1:133414‑M2/283‑301 (MQ=255)`  
              `gagatgtgtataagggacatcactaaaggagacacaccattttcaaacttattaagaagttaggccaactgctggttcgtatgtacaacgtggaagccaagcgactgaacgatgaggctcgtaaagaggccacacagtcacgcgctctggcgattcgctccaacgaactggctgacagtgcatccactaaagttaccgaggctgcccgtgtggcaaaccaagctcaacagctttccaaattctttgagtaatcaaacaggagaaaccattatgATCGTTTCTGACATCGAAGCTAACGccc                                                                                                                                                                                                                                                                                   <  1:248703‑M2/28‑1 (MQ=255)`  
              `agtcccattctaatacgactcactaaaggagacacaccatgttcaatctgattaagaagttaggccaactgctggtgcgtatgtacaacgtggaagccaagcgactgaacgatgaggctcgtaaagaggccacacagtcacgcgctctggcgattcgctccaacgaactggctgacagtgcatccactaaagttaccgaggctgcccgtgtggcaaaccaagctcaacagctttccaacttctttgagtaatcaaacaggagaaaccattatgATCGTTTCTGACATCGAAGCTAACGccc                                                                                                                                                                                                                                                                                   <  1:198034‑M2/28‑1 (MQ=255)`  
               `gtcccattctaatacgactcactaaaggagacacaccatgttcaaactgattaagaagttaggccaactgctggttcgtatgtacaacgtggaagccaagcgactgaacgatgaggctcgtaaagaggccacacagtcacgcgctctggcgattcgctccaacgaactggctgacagtgcatccactaaagttaccgaggctgcccgtgtggcaaaccaagctcaacagctttccaaattctttgagtaatcaaacaggagaaatcaatatgATCGTTTCTGACATAGAAGCTAACGCCct                                                                                                                                                                                                                                                                                  >  1:161463‑M2/273‑301 (MQ=255)`  
                  `ccattctaatacgactcactaaaggagacacaccatgttcaaactgattaagaagttaggccaactgctggttcgtatgtacaacgtggaagccaagcgactgaacgatgaggctcgtaaagaggccacacagtcacgcgctctggcgattcgctccaacgaactggctgacagtgcatccactaaagttaccgaggctgcccgtgtggcaaaccaagctcaacagctttccaaattctttgagtaatcaaacaggagaaatcaatatgATCGTTTCTGACATCGAAGCTAACGCCCTCtt                                                                                                                                                                                                                                                                               >  1:475949‑M2/270‑301 (MQ=255)`  
                     `ttctaatacgactcactaaaggagacacaccatgttcaaacttattaagaagttaggccaactgctggttcgtatgtacaacgtggaagccaagcgactgaacgatgaggctcgtaaagaggccacacagtcacgcgctctggcgattcgctccaacgaactggctgacagtgcatccactaaagttaccgaggctgcccgtgtggcaaaccaagctcaacagctttccaaattctttgagtaatcaaacaggagaaatcaatatgATCGTTTCTCACATCGAACCTAACGCCCTCTTaga                                                                                                                                                                                                                                                                            >  1:379451‑M2/267‑301 (MQ=255)`  
                     `ttctaatacgactcactaaaggagacacaccatgttcaaactgattaagaagttaggccaactgctggttcgtatgtacaacgtggaagccaagcaactgaacgatgaggctcgtaaagaggccacacagtcacgcgctctggcgattcgctccaacaaactggctgaccgtgcatccaataaagttacagaggctgcccgtgtggacaaccaagccaaacagctttccaaatcttttgaataatcaaacaggagcaaccattatgATCCTTTCTTACATCAAAGCTAACGCCCTCttaaa                                                                                                                                                                                                                                                                            >  1:494797‑M2/267‑299 (MQ=255)`  
                     `ttctaatacgactcactaaaggagacacaccatgttcaaactgattaagaagttaggccaaatgctggttcgtatgtacaacgtggaagccaatcgactgaacgatgaggctcgtaaagaggccacacagtcacgcgctctggcgattcgctccaacgaactggctgacagtgcatccactaaagttaccgaggctgcccgtgtggcaaaccacgctcaaaagctttccaaattctttgagtaatcaaacaggaaaaaccattatgATCGTTTCTGCCATCGAAGCTAacgccctcctgtc                                                                                                                                                                                                                                                                            >  1:54298‑M2/267‑296 (MQ=255)`  
                     `ggcgtaatcagcaaggcgatatgcctaaccttgtcctcgttcgtattctcaagtgccgctttactggtgatactggtatcgctggctacatggaatacaacaaggaaaccggatggcttgaacaatcaagttactcaggggaagaagagtcacactcagagtcaacagactggtccaacgacactgacttctgacaggattcttgaCAGTTGTTTCATATGAAGAGATTGTTAAGTCACGATAATC‑AATAGGAGAAATCAATATGATCGTTTCTGACATCGAAGCTAACGCCCTCTTagag                                                                                                                                                                                                                                                                           <  1:1793‑M2/95‑1 (MQ=255)`  
                      `tttaatacgactcactaaaggatacacaccatgttccaactgattaagaagttaggccaactgctggttcttatgtacaacgtggaagccaagcgactgaacgatgaggctcgtaaagaggccacacagtcacgcgctctggcgattcgctccaacgaactggctgacagtgcatccactaaagttaccgaggctgcccgtgtggcaaaccaagctcaacagctttccaaattctttgagtaatcaaacaggagaaaccattatgATCGTTTCTGACATCGAAGCTAACGCCCTCTTagag                                                                                                                                                                                                                                                                           <  1:63756‑M2/36‑1 (MQ=255)`  
                      `tctaatacgactcactaaaggagacacaccatgttcaaactgattaagaagttaggccaactgctgggtcgtatgtacaacgtggaagccaagcgactgaacgatgaggctcgtaaagaggccacacagtcacgcgctctggcgattcgctccaacgaactggctgacagtgcatccactaaagttaccgaggctgcccgtgtggcaaaccaagctcaacagctttccaaattctttgagtaatcaaacaggagaaatcaatatgATCGTTTCTGACATCGAAGCTAACGCCCTCTTagag                                                                                                                                                                                                                                                                           <  1:193381‑M2/36‑1 (MQ=255)`  
                      `tataagcgacagcactaaaggagacacaccatgttcaaactgattaagaagttaggccaactgctggttcgtatgtacaacgtggaagccaagcgactgaacgatgaggctcgtaaagaggccacacagtcacgcgctctggcgattcgctccaacgaactggctgacagtgcatccactaaagttaccgaggctgcccgtgtggcaaaccaagctcaacagctttccaaattcttttagtaatcaaacaggagaaaccattatgATCGTTTCTGACATCGAAGCTAACGCCCTCTTagag                                                                                                                                                                                                                                                                           <  1:451933‑M2/36‑1 (MQ=255)`  
                      `agatgtgtataagagacagggagacacaccatgttcaaactgattaagaagttaggccaactgctggttcgtatgtacaacgtggaagccaagcgactgaacgatgaggctcgtaaagaggccacacagtcacgcgctctggcgattcgctccaacgaactggctgacagtgcatccactaaagttaccgaggctgcccgtgtggcaaaccaagctcaacagctttccaaattctttgagtaatcaaacaggagaaaccattatgATCGTTTCTGACATCGAAGCTAACGCCCTCTTagag                                                                                                                                                                                                                                                                           <  1:156501‑M2/36‑1 (MQ=255)`  
                             `cgactcactaaaggagacacaccatgttcaaactgattaagaagttaggccaactgctggttcgtatgtacaacgtggaagccaagcgactgaacgatgaggctcgtaaagaggccacacagtcacgcgctctggcgattcgctccaacgaactggttgacagtgcatccactaaagttaccgaggctgcccgtgtggcaaaccaagctcaacagctttccaaattctttgagtaatcaaacatgagaaaccattatgATCGTTTCTGAAATCGAAGCTAAcgccctcttagctgtctctt                                                                                                                                                                                                                                                                    >  1:26939‑M2/259‑292 (MQ=255)`  
                              `gactcactaaaggagacacaccatgttcaaactgattaagaagttaggccaactgctggttcgtatgtacaacgtggaagccaagcgactgaacgatgaggctcgtaaagaggccacacagtcacgcgctctggcgattcgctccaacgaactggctgacagtgcatccactaaagttaccgaggctgcccgtgtggcaaaccaagctcaacagctttccaaattctttgagtaatcaaacaggagaaaccattatgATCGTTTCTGACATCGAAGCTAACGCCCTCTTAGAGAGCGTCAc                                                                                                                                                                                                                                                                   <  1:238754‑M2/44‑1 (MQ=255)`  
                                `ggagatgtttataaggaacagtgttcaaacgtattaagaagttaggccaactgctggttcgtatgtacaacgtggaagccaagcgactgaacgatgaggctcgtaaagaggccacacagtcacgcgctctggcgattcgctccaacgaactggctgacagtgcatccactaaagttaccgaggctgcccgtgtggcaaaccaagctcaacagctttccaaattctttgagtaatcaaacaggagaaaccattatgATCGTTTCTGACATCGAAGCTAACGCCCTCTTAGAGAGCGTCACTa                                                                                                                                                                                                                                                                 <  1:126260‑M2/46‑1 (MQ=255)`  
                                `aatgtgtataagaaacagccatgttcaaactgattaagaagttaggccaactgctggttcgtatgtacaacgtggaagccaagcgactgaacgatgaggctcgtaaagaggccacacagtcacgcgctctggcgattcgctccaacgaactggatgacagtgcatccactaaagttaccgaggctgcccgtgtggcaaaccaagctcaacagctttccaaattctttgagtaatcaaacaggagaaaccattatgATCGTTTCTGACATCGAAGCTAACGCCCTCTTAGAGAGCGTCACTa                                                                                                                                                                                                                                                                 <  1:112744‑M2/46‑1 (MQ=255)`  
                                   `cactaaaggagacacaccatgttcaaactgattaagaagttaggccaactgctggttcgtatgtacaacgtggaagccaatcgactgaacgatgaggctcgtaaataggccacacagtcacgcgctctggcgattcgctccaacgaactggctgacagtgcatccactacagttacctaggctgcccgtgtggcaaaccaagctcaacagctttccaaattctttgagtaatcaataggagaaatcaatatgATCGTTTCTGACATCGAAGCTAACGCACACTTAGAGATTGTCACTAAGt                                                                                                                                                                                                                                                              >  1:398287‑M2/253‑301 (MQ=255)`  
                                     `aagaggagacacaccatgttcaaactgattaagaagttaggccaactgctggttcgtatgtacaacgtggaagccaagcgactgaacgatgaggctcgtaaagaggccacacagtcacgcgctctggcgattcgctccaacgaactggctgacagtgcatccactaaagttaccgaggctgcccgtgtgtcaaaccaagctcaacagctttccaaattctttgagtaatcaaacaggagaaaccattatgATCGTTTCTGACATCGAAGCTAACGCCCTCTTAGAGAGCGTCACTAAGTTc                                                                                                                                                                                                                                                            <  1:58889‑M2/51‑1 (MQ=255)`  
                                        `aggagacacaccatgttcaaactgattaagaagttaggccaactgctggttcgtatgtacaacgtggaagccaagcgactgaacgatgaggctcgtaaagaggccacacagtcacgagctctggcgattcgctccaacgaactggctgacagtgcatccactaaagttaccgaggctgcccgtgtggcaaaccaagctcaacagctttccaaattctttgagtaatcaaacaggagaaaccattatgATCGTTTCTGACATCGAAGCTAACGCCCTCTTAGAGAGCGTCACTAAGTTCCAc                                                                                                                                                                                                                                                         >  1:339852‑M2/248‑301 (MQ=255)`  
                                            `gacacaccatgttcaaactgattaagaagttaggccaactgctggttcgtatgtacaacgtggaagccaagcgactgaacgatgaggctcgtaaagaggccacacagtcacgcgctctggctattcgctccaacgaactggctgacagtgcatccactaaagttaccgaggctgcccgtgtggcaaaccaagctcaacagctttccaaattctttgagtaatcaaacaggagaaaccattatgATCGTTTCTGACATCGAAGCTAACGCCCTCTTAGAGAGCGTCACTAAGTTCCACTGCg                                                                                                                                                                                                                                                     >  1:8185‑M2/244‑301 (MQ=255)`  
                                               `acaccatgtgcaaactgattaagaagttaggccaactgctggttcgtatgtacaacgtggaagccaagcgcatgaacgatgaggctcgtaaagaggccacacagtcacgcgctctggcgattcgctccaacgaactggctgacagtgcaaccactaaagttaccgaggctgcccgtgtggcaaaccaagctcaacagctttccaaattctttgagtaatcaaacaggagaaatcaatatgATCGTTTCTGACATCGAAGCTAACGCCCTCTTAGAGAGCGTCACTAAGTTCCACTGCgggg                                                                                                                                                                                                                                                  <  1:371334‑M2/61‑1 (MQ=255)`  
                                                  `ccatgttcaaactgattaagaagttaggccaactgctggttcgtatgtacaacgtggaagccaagcgactgaacgatgaggctcgtaaagaggccacacagtcacgcgctctggcgattcgctccaacgaactggctgacagtgcatccactaaagttaccgaggctgcccgtgtggcaaaccaagctcaacagctttccaaattctttgagtaatcaaacaggagaaaccattatgATCGTTTCTGACATCGAAGCTAACGCCCTCTTAGAGAGCGTCACTAAGTTCCACTGCGGGGTTa                                                                                                                                                                                                                                               >  1:443865‑M2/238‑301 (MQ=255)`  
                                                   `caggttcaaactgattaagaagttaggccaactgatggttcgtatgtacaacgtggaagccaagcgactgaacgatgaggctcgtaaagaggccacacagtcacgcgctctggcgattcgctccaacgaactggatgacagtgcatccactaaagttaccgaggctgcccgtgtggcaaaccaagctcaacagctttccacattctttgagtaatcaaacaggaagaatcaatatgATCGTTTCTGACATCGAATCTAACGCCCTCTTATAGAGCATCACTAAGTTCCACAGCGGGGTTAt                                                                                                                                                                                                                                              >  1:294391‑M2/237‑301 (MQ=255)`  
                                                     `ttttcaaactgattatgaagttaggccaactgctggttcgtatgtacaaagtggaagccaagctactgaacgatgaggctcgtaaagaggccacacagtcacgctctctggctattcgctccaacgaactggctgacagtgcatccactaaagttaccgaggctgcccgtgttgcaaaccaagctcaacagctttccaaattctttgagtaatcaaacatgagaaaccattatgATCGTTTCTGACATCGAAGCTAACGCCCTCTTAGAGAGCGTCACTAAGTTCCACTGCGGGGTTATCt                                                                                                                                                                                                                                            <  1:86750‑M2/67‑1 (MQ=255)`  
                                                      `gttcaaactgattaagaagttaggccaactgctggttcgtatgtacaacgtggaagccaagcgactgaacgatgagtctcgtaaagaggccacacagtcacgcgctctggcgattcgctccaacgaactggctgacagtgcatccactaaagttaccgaggctgcccgtgtggcaaaccaagctcaacagctttccaaattctttgagtaatcaaacaggagaaaccattatgATCGTTTCTGACATCGAAGCTAACGCCCTCTTAGAGAGCGTCACTAAGTTCCACTGCGGGGTTATCTa                                                                                                                                                                                                                                           >  1:184168‑M2/234‑301 (MQ=255)`  
                                                          `aaactgattaagaggttaggccaactgctggttcggatgtacaacgtggaagccaagcgactgaacgatgaggctcgtaaagaggccacacagtcacgcgctctggcgattcgctccaacgaactggctgacagtgcatccactaaagttaccgaggctgcccgtgtggcaaaccaagctcaacagctttccaaattctttgagtaatcaaacaggagaaaccattatgATCGTTTCTGACATCGAAGCTAACGCCCTCTTAGAGAGCGTCACTAAGTTCCACTGCGGGGTTATCTACGac                                                                                                                                                                                                                                       <  1:233935‑M2/72‑1 (MQ=255)`  
                                                             `ctgattaagaagttaggccaactgctggttcgtatgtacaacgtggaagccaagcgactgaacgatgaggctcgtaaagaggccacacagtcacgcgctctggcgattcgctccaacgaactggctgacagtgcatccactaaagttaccgaggctgcccgtgtggcaaaccaagctcaacagctttacaaattctttgagtaatcaaacaggagaaaacattatgATCGTTTCTGACATCGAAGCTAACGCCCTCTTATAGATCGTCCCTAAGTTCCACTGCGGGCTTATCTacgacctt                                                                                                                                                                                                                                    >  1:334019‑M2/227‑298 (MQ=255)`  
                                                               `gattaagaagttcggccaactgctggttcgtatgtacaacgtggaagccaagcgactgaacgatgaggctcgtaaagaggccacaaagtcacgcgctctggcgattcgctccaacgaactggctgacagtgaatccactaaagttaccgaggttgcccgtgtggcaaaccaagctcaaccgctatcccacttctttgcgtaatctaaaaggagaaaccattatgATCGTTTCTGACATCGAAGCTAACGCCCTCTTAGAGAGCGTCACTAAGTTCCACTGCGGGGTTATCTACgaccactg                                                                                                                                                                                                                                  >  1:416176‑M2/225‑300 (MQ=255)`  
                                                                 `attctcaagtgccgatttactggtgatactggtatcgctggctacatggaatacaacaaggaaaccggatggcttgaaccatcaagttactcaggggaagaagagtcacactcagagtcaacagactggtccaacgacactgacttctgacaggattcttgaCAGTTGTTTCATATGAAGAGATTGTTAAGTCACGATAATC‑AATAGGAGAAATAAATATGATCGTTTCTGACATCGAAGCTAACGCCCTCTTAGAGAGCGTCACTAAGTTCCACTGCGGGGTtatctacgacctgtctct                                                                                                                                                                                                                               >  1:95196‑M2/163‑293 (MQ=255)`  
                                                                    `agaagttaggccaactgctggttcgtatgtacaacgtggaagccaagcgactgaacgatgaggctcgtaaagaggccacacagtcacgcgctctggcgattcgctccaacgaactggctgacagtgcatccactaaagttaccgaggctgcccgtgtggcaaaccaagctcaacagctttccaaattctttgagtaatcaaacaggagaaatcaatatgATCGTTTCTGACATCGAAGCTAACGCCCTCTTAGAGAG‑GTCACTAAGTTCCACTGCGGGGTTATCTACGACTACTCCACCGc                                                                                                                                                                                                                            <  1:471144‑M2/82‑1 (MQ=255)`  
                                                                      `aagttatgccaactgctggttcgtatgtacaacgtggaagccaagcgactgaacgatgaggctcgtaaagaggccacacagtcacgcgctctggcgattcgctccaacgaactggctgacagtgcatccactaaagttaccgaggctgcccgtgtggcaaaccaagctcaacagctttccaaattctttgagtaatcaaacaggagaaaccattatgATCGTTTCTGACATCGAAGCTAACGCCCTCTTAGAGAGCGTCACTAAGTTCCACTGCGgggttatctacgacctgtctcttata                                                                                                                                                                                                                           >  1:312855‑M2/218‑289 (MQ=255)`  
                                                                             `gggTTAAGGTTGACATGGGAGTTGTTGGTGGATACGGCTACGTCCGCTCAGTTAGTGGCAAATATGCACAAGTGTCATACATCACAGGTGTTACTCCACGCGGTGCAATCGTTGCCGATAAGACCAACATGATTCACACAGGTTTCTTGACAGTTGTTTCATATGAAGAGATTGTTAAGTCACGATAATC‑AATAGGATAAATCAATATGATCGTTTCTGACATCGAAGCTACCGCCCTCTTAGAGAGCGTCACTAAGTTCCACTGCGGGGTTATCTACGACTACTCCACCGCTGAGTACGt                                                                                                                                                                                                                   >  1:272662/1‑301 (MQ=255)`  
                                                                               `aaactgcttgttcgtatgtacaacgtggaagccaagcgactgaacgatgaggctcgtaaagaggccacacagtcactcgctctggcgattcgcgccaacgaactggctgacagtgcatccactaaagttaccgaggctgcccgtgtgtcaaaccaagctcaacagctttccaaattctttgagtaatcaaacaggagaaaccattatgATCGTTTCGGACATCGAAGCTAACGCCCTCTTAGAGAGCGTCACTAAGTTCCACTGCGGGGTTATCTACGACTACTCCACCGCTGAGTACGTa                                                                                                                                                                                                                  <  1:166288‑M2/93‑1 (MQ=255)`  
                                                                                  `ctgctggttcgtatgtacaacgtggaagccaagcgactgaacgatgaggctcgtaaagaggccacacagtcacgcgctctggcgattcgctccaacgaactggctgacagtgcatccactaaagttaccgaggctgcccgtgtggcaaaccaagctcaacagctttccaaattctttgagtaatcaaacaggagaaaccattatgATCGTTTCTGACATCGAAGCTAACGCCCTCTTAGAGAGCGTCACTAAGTTCCACTGCGGGGTTATCTACGACTACTCCACCGCTGAGTACGTAAGc                                                                                                                                                                                                               >  1:73333‑M2/206‑301 (MQ=255)`  
                                                                                  `ctgctggttcgtatgtacaacgtcgaagccaagcgactgaacgatgaggctcgtaaagaggccacacagtaacgcgctctggcgattcgctccaacgaactggctgacagtgcatccactaaagttaccgaggagtcccgtgtggcaaaccaagctaaacatctttccaaattctatgagtaatcaaacaggagaaaccattatgATCGTTTCTGACATAGAAGCTAACGCCCTCTTAGAGAGCGTCACTAAGTTCCACTGCGGGGTTATCTACGACTACTCCACCGCTGAGTACGTAAGc                                                                                                                                                                                                               <  1:304326‑M2/96‑1 (MQ=255)`  
                                                                                  `ctgctggttcgtatggacaacgtggaagccaagcgactgaacgatgaggctcgtaaagaggccacacagttacgcgctctggcgattcgctccaacgaactggctgacagtgcatccactaaagttaccgaggctgcccgtgtggcaaaccaagctcaacagctttccaaattctttgaagaatcaaacaggagaaatcaatatgATCTTTTCTGACATCGAAGCTAACGCCCTCTTAGAGAGCGTCACTAAGTTACACTGCGGGGTTATCTACGACTACTCCACCGCTGAGTACGTAAGc                                                                                                                                                                                                               <  1:318204‑M2/96‑1 (MQ=255)`  
                                                                                  `aagagacagcgtatgtacaacgtggaagccaagcgactgaacgatgaggctcgtaaagaggccacacagtcacgcgctctggcgattcgctccaacgaactggctgacagtgcatccactaaagttaccgaggctgcccgtgtggcaaaccaagctcaacagctttccaaattctttgagtaatcaaacaggagaaatcaatatgATCGTTTCTGACATCGAAGCTAACGCCCTCTTAGAGAGCGTCACTAAGTTCCACTGCGGGGTTATCTACGACTACTCCACCGCTGAGTACGTAAGc                                                                                                                                                                                                               <  1:289995‑M2/96‑1 (MQ=255)`  
                                                                                       `ggttcgtatgtacaacgtggaagccaagcgactgaactatgaggctcgtaaagaggccccacagtcacgcgctctggcgattcgctccaacgaactggctgacagttcatccactaaagttaccgaggctgcccgtgtggcaaaccaagctcaacagctttccaaattctttgagtaatcaaacaggagaaaccattatgATCGTTTCTGACATCGAAGCTAACGCCCTCTTAGAGAGCGTCACTAAGTTCCACTGCGGGGTTATCTACGACtcctcccccgcttagctgtctcttataca                                                                                                                                                                                                          >  1:434591‑M2/201‑287 (MQ=255)`  
                                                                                             `tatgtacaacgtggaagccaagcgactgaacgatgaggctcgtaaagaggccacacagtcacgcgctctggcgattcgctccaacgaactggctgacagtgcatccactaaagttaccgaggctgcccgtgtggcaaaccaagctcaacagctttccaaattctttgagtaatcaaacagcagaaaccattatgATCGTTTCTGACATCGAATCTAACGCCCTCTTAGAGAGCGTCACTAAGTTCCACTTCTGGGTTATCTACGCCTACTCCACCGCTGATTCCGTACGCTACCTTCCGAg                                                                                                                                                                                                    >  1:30805‑M2/195‑301 (MQ=255)`  
                                                                                               `tgtacaacgtggaagccaagcgactgaacgatgatgctcgtaaagaggccacacagtcacgcgctctggcgattcgctccaactaaatggctgacagtgcatccactaaagttaccgaggctgcgcgtgtggcaaaccaagctcaacagctttccaaattctttgagtaatcaaacaggagaaaccattatgATCGTTTCTTACATCGAAGCTAACGCCCTCTTAGATAGCTTCACTAAGTTCCACTGCGGGGTTATCTACGACTACTCCACCGCTGAGTACGTAAGCTACCGTCCGAGTg                                                                                                                                                                                                  <  1:510045‑M2/109‑1 (MQ=255)`  
                                                                                                              `ccaagcgactgaacgatgaggctcgtaaagaggccacacagtcacgcgctctggcgattcgctccaacgaactggctgacagtgcatccactaaagttaccgaggctgcccgtgtggcaaaccaagctcaacagctttccaaattctttgagtaatcaaacaggagaaaccattatgATCGTTTCTGACATCGAAGCTAACGCCCTCTTAGAGAGCGTCACTAAGTTCCACTGCGGGGTTATCTACGACTACTCCACCGCTGAGTACGTAAGCTACCGTCCGAGTGACTTCGGTGCGTATc                                                                                                                                                                                   <  1:220518‑M2/124‑1 (MQ=255)`  
                                                                                                              `atggaatacaacaaggaaaccggatggcttgaaccatcaagttactcaggggaagaagagtcacactcagagtcaacagactggtccaacgacactgacttctgccaggattcttgaCAGTTGTTTCATATGAAGAGATTGTTAAGTCACGATAATC‑AATAGGAGAAATCAATATGATCGTTTCTGACATCGAAGCTAACGCCCTCTTAGAGAGCTTCACTAAGTTCCACTGCGGGGTTATCTACGACTACTCCACCGCTGAGTACTTACGCTACCGTCCGAGTTACTTCGGTGCGTATCt                                                                                                                                                                                  >  1:48727‑M2/118‑301 (MQ=255)`  
                                                                                                                  `gcgactgaacgatgaggcccgtaaagaggccacacagtcacgcgctctggcgattcgctccaacgaactggctgacagtgcatccactaaagttaccgaggctgcccgtgtggcaaaccaagctcaacagctttccaaattctttgagtaatcaaacaggagaaaccattatgATCGTTTCTGACATCGAAGCTAACGCCCTCTTAGAGAGCGTCACTAAGTTCCACTGCGGGGTTATCTACGACTACTACACCGCTGAGTACGTAAGCTACCGTCCGAGTGACTTCGGTGCGTATCTGGa                                                                                                                                                                               <  1:121570‑M2/128‑1 (MQ=255)`  
                                                                                                                      `ctgaacgatgaggctcgtaaagaggccacacagtcacgcgctctggcgattcgctccaacgaactggctgacagtgcatccactaaagttaccgaggctgcccgtgtggcaaaccaagctcaacagctttccaaattctttgagtaatcaaacaggagaaaccattatgATCGTTTCTGACATCGAAGCTAACGCCCTCTTAGAGAGCGTCACTAAGTTCCACTGCGGGGTTATCTACGACTTCTCCACCGCTGAGTACGTAAGCTACCGTCCGAGTGACTTCGGTGCGTATCTGGAtgcc                                                                                                                                                                           >  1:173567‑M2/170‑300 (MQ=255)`  
                                                                                                                        `gaacgatgaggctcgtaaagaggccacacagttacgcgctctggcgattcgctccaacgaactggctgacagtgcatccactaaagttaccgaggctgcccgtgtggcaaaccaagctcaacagctttccaaattctttgagtaatcaaacaggagaaaccattatgATCGTTTCTGACATCGAAGCTAACGCCCTCTTAGAGAGCGTCACTAAGTTCCACTGCGGGGTTATCTACGACTACTCCACCTCTGAGTACGTAAGCTACCGTCCGAGGGACTTCGGTGCGTATCTGGATGCGCt                                                                                                                                                                         >  1:426781‑M2/168‑301 (MQ=255)`  
                                                                                                                               `aaggctccaaaagagtccacatagtaacgcggtcaggcgattcgctccaacaaactggctgacagtgcatccactaaagttaccgaggctgcccgtgtgtcaaacaaagctaaacagctttccaaattcgatgagtaatcaaacaggagaaatcaatatgATCGTTTCTGACATCGAAGCTAACGCCCTCTTAGAGAGCGTCACTAAGTTCCACTGCGGGGTGATCTACGACTACTCCACCGCTGAGTACGTAAGCTACCGTCCGAGTGACTTCGGTGCGTATCTGGATGCGCTGGAAGcc                                                                                                                                                                  <  1:353444‑M2/141‑1 (MQ=255)`  
                                                                                                                                  `gctcgtaaagaggccacacagtcacgcgctctggcgattcgctccaacgaactggctgacagtgcatccactaaagttaccgaggctgcccgtgtggcaaaccaagctcaacagctttccaaattatttgaggaatcaaacaggagaaaacattatgATCGTTTCTGAGATAGAAGTTAACGCACTATTAGAGAGCGTCACTAAGTTCCACTTCGGGGTTATCTACGACTACTCCACCGCTGAGTACGTAAGCTACCGTCCGCGCGACTCAGGTGCGTATCTGGATGCGCGTGaagccttt                                                                                                                                                               >  1:299369‑M2/158‑298 (MQ=255)`  
                                                                                                                                             `ggccacacactcacgcgctctggccattcgctccaccgacctggctgacagtgcatccactaaagtttccgcggctgccagtgtggcaaaccaagctcaccagctttccatatgctttgagtaatcaagcaggagagatcaatatgATCGTTTCTGACATCGAAGCTAACGCCCTCTTAGAGAGCGTCACTAAGTTCCACTGCGGGGTTATATACGACTACTCCACCGATTAGTACGTAAGCTACCGTCCGAGTGACTTCGGTGCGTATCTGGATGCGCTGGAAGCCGAGGTTGCACGAgg                                                                                                                                                    <  1:362441‑M2/155‑1 (MQ=255)`  
                                                                                                                                             `aaccatcaagttactcaggggaagaagagtcacactcagagtcaacagactggtccaacgacactgacttctgacaagattcttgaCAGTTGTTTCATATGAAGAGATTGTTAAGTCACGATAATC‑AATAGGATAAATCAATCTGATCGTTTCTGACATCGAAGCTAACGCCCTCTTAGAGAGCGTCACTAAGTTCCACTGCGGGGTTATCTACGACTACTCCACCGCTGAGTACGTAAGCTACCGTCCGAGTGACTTCGGTGCGTATCTGGATGCGCTGGAAGCCGAGGTTGCACGAGGc                                                                                                                                                   <  1:349384‑M2/215‑1 (MQ=255)`  
                                                                                                                                                  `agcagtcacgcgcgctggcgattcgctccaacgaactggctgacagtgcatccactaaagttaccgaggctgcccgtgtggcaaaccaagctcaacagctttccaaattctttgagtaatcaaacaggagaaatcaatatgATCGTTTCTGACATCGAAGCTAACGCCCTCTTAGAGAGCGTCACTAAGTTCCACTGCGGGGTTATCTACGACTACTCCACCGCTGAGTACGTAAGCTACCGTCCGAGTGACTTCGGTGCGTATCTGGATGCGCTGGAAGCCGAGGTTGCACGAGGCGGTc                                                                                                                                               <  1:91271‑M2/160‑1 (MQ=255)`  
                                                                                                                                                   `aaggtcacgcgctttggcgattcgctccaaccaactggctgacagtgcatccactaaagttaccgaggctgcccgtgttgcaaaccaagctcaacagctttccaaatgctttgagtaatcaaacaggagaaaccattatgATCGTTTCTGACATCGAAGCTAACGCCCTCTTAGAGAGCGTCACTAAGTTCCACTGCGGGGTTATCTACGACTACTCCACCGCTGAGTACGTAAGCTACCGTCCGAGTGACTTCGGTGCGTATCTGGATGCGCTGGAAGCCGAGGTTGCACGAGGCGGTCt                                                                                                                                              <  1:258207‑M2/161‑1 (MQ=255)`  
                                                                                                                                                    `aagttactcaggggaagaagagtcacactcagagtcaacagactggtccaacgacactgacttctgacaggattcttgaCAGTTGTTTCATATGAAGAGATTGTTAAGTCACGATAATC‑AATAGGAGAAATCAATATGATCGTTTCTGACATCGAAGCTAACGCCCTCTTAGAGAGCGTCACTAAGTTCCACTGCGGGGTTATCTACGACTACTCCACCGCTGAGTACGTAAGCTACCGTCCGAGTGACTTCGGTGCGTATCTGGATGCGCTGGAAGCCGAGGTTGCACGAGGCGGTCTTa                                                                                                                                            >  1:448281‑M2/80‑301 (MQ=255)`  
                                                                                                                                                      `gtcacgcgctctggcgattcgctccaacgaactggctgacagtgcatccactaaagttaccgaggctgcccgtgtggcaaaccaagctcaacagctttccaaattctttgagtaatcaaacaggagaaaccattatgATCGTTTCTGACATCGAAGCTAACGCCCTCTTAGAGAGCGTCACTAAGTTCCACTGCGGGGTTATCTACGACTACTCCACCGCTGAGTACGTAAGCTACCGTCCGAGTGACTTCGGTGCGTATCTGGATGCGCTGGAAGCCGAGGTTGCACGAGGCGGTCTTAt                                                                                                                                           >  1:389050‑M2/138‑301 (MQ=255)`  
                                                                                                                                                      `gtcacgcgctctggcgattcgctccaacgaactggctgacagtgcatccactaaagttaccgaggctgcccgtgtagcaaaccaagctcaacagctttccaaattctttgagtaatcaaacaggagaaatcaatatgATCGTTTCTGACATCGAAGCTAACGCCCTCTTAGAGAGCGTCACTAAGTTCCACTGCGGGGTTATCTACGACTACTCCACCGCTGAGTACGTAAGCTACAGTCCGAGTGACTTCGGTGCGTATCTGGATGCGCTGGAAGCCGAGGTTGCACGAGGCGGTCTTAt                                                                                                                                           <  1:463246‑M2/164‑1 (MQ=255)`  
                                                                                                                                                      `gtcacgcgctctggcgattcgctccaacgaactggctgacagtgcatccactaaagttaccgaggctgcacgtgtggcaaaccaagctcaacagctttccaaattctttgagtaatcaaacaggagaaaccattatgATCGTTTCTGACATCGAAGCTAACGCCCTCTTAGAGAGCGTCACTAAGTTCCACTGCGGGGTTATCGACGACTACTCAACCGCTGAGTACGTAAGCTAACGTCAGAGTGACTTCGGTTAGAAACAGGATGGGATGGAAGCCGAGGTTGAACGAGACGGTCTtag                                                                                                                                           >  1:383846‑M2/138‑300 (MQ=255)`  
                                                                                                                                                      `gtcacgcgctctggcgattcgctccaacgaactggctgacagtgcatccactaaagttacagaggctgcccgtgtggcaaaccaagctcaacagctttccaaattctttgagtaatcaaacaggagaaatcaatatgATCGTTTCTGACATCGAAGCTAACGCCATCTTCGAGAGCGTCACTAAGTTCCACTGCGGGGTTATCTACGACTACTCAACCGCTGAGTACTTAAGCTACCGTCCGAGTGACTTCGGTGCGTATCTGGATGCGCTGGAAGCCGAGGTTGAACGAGGCGGTCTTAt                                                                                                                                           >  1:344868‑M2/138‑301 (MQ=255)`  
                                                                                                                                                         `actcaggggaagaagagtcacactcagagtcaacagactggtccaacgacactgacttctgacaggattcttgaCAGTTGTTTCATATGAAGAGATTGTTAAGTCACGATAATC‑AATAGGAGAAATCAATATGATCGTTTCTGACATCGAAGCTAACGCCCTCTTAGAGAGCGTCACTAAGTTCCACTGCGGGGTTATCTACGACTACTCCACCGCTGAGTACGTAAGCTACCGTCCGAGTGACTTCGGTGCGTATCTGGATGCGCTGGAAGCCGAGGTTGCACGAGGCGTGCTTattgct                                                                                                                                       >  1:48138‑M2/75‑299 (MQ=255)`  
                                                                                                                                                            `acaggtaagaagattcacactgatagtcagcagactggtcccacgacattgatttctgtcaggattattgaCATTTGTTTAATATGAAGAGATTGTTAAGTCACGTTAATC‑AATAGGAGAAATCAATATGATCGTTTCTGACATCGAAGCTAACGCCCTCTTAGATAGCGTCACTAAGTTCCACTGCGGGGTTATCTACGACTACTCCACCGCTGAGTACGTAAGCTACCGTCCGAGTGACTTCGGTGCGTATCTGGATGCGCTGGAAGCCGAGGTTGCACGAGGCGGTCTTATTGTGTTc                                                                                                                                    <  1:67999‑M2/230‑1 (MQ=255)`  
                                                                                                                                                              `ggggaagaagagtcacactcagaatcaacagactggtccaacgacactgacttctgacaggattcttgaCAGTTGTTTCATATGAAGAGATTGTTAAGTCACGATAATC‑AATAGGAGAAATCAATATGATCGTTTCTGACATCGAAGCTAACGCCCTCTTAGAGAGCGTCACTAAGTTCCACTGCGGGGTTATCTACGACTACTCCACCGCTCAGTACGTAAGCTACCGTCCGAGTGACTTCGGTGCGTCTCTGGATGCGCTGGAAGCCGAGGTTGCACGAGGCGGTCTTATTGTGTTCca                                                                                                                                  >  1:214170‑M2/70‑301 (MQ=255)`  
                                                                                                                                                              `ggggaagaagagtcacaatcagagtcaacagactggtccaacgacactgacttctgacaggattcttgaCAGTTGTTTCATATGAAGAGATTGTTAAGTCACGATAATA‑AATAGGAGAAATCAATATGATCGTTTCTGACATCGAAGCTAACGCACTCTTAGAGAGCGTCACTAAGTTCCACTGCGGGGTTATCTAAGACTACTCCACAGCTGAGTACGTAAGCTACCGTCCGAGTGACTTCGGTGCGTATCTGGATGCGCAGGAAGCTGAGGTTGCACGAGGCGGTCGTATTGTGTGCca                                                                                                                                  >  1:78392‑M2/70‑301 (MQ=255)`  
                                                                                                                                                                    `gtgtTACTCCACGCGGTGCAATCGTTGCCGATAAGACCAACATGATTCACACAGGTTTCTTGACAGTTGTTTCATATGAAGAGATTGTTAAGTCACGATAATC‑AATAGGAGAAACCATTATGATCGTTTCTGACATCGAAGCTAACGCCCTCTTAGAGAGCGTCACTAAGTTCCACTGCGGGGTTATCTACGACTACTCCACCGCTGAGTACGTAAGCTACCGTCCGAGTGACTTCGGTGCGTATCTGGATGCGCTGGAAGCCGAGGTTGCACGAGGCGGTCTTCTTGTGTTCCACAACgg                                                                                                                            >  1:203284/1‑301 (MQ=255)`  
                                                                                                                                                                         `gtcacactcagagtcaacagactggtccaacgacactgacttctgacaggattcttgaCAGTTGTTTCATATGAAGAGATTGTTAAGTCACGATAATC‑AATAGGAGAAATCAATATGATCGTTTCTGACATCGAAGCTAACGCCCTCTTAGAGAGCGTCACTAAGTTCCACTGCGGGGTTATCTACGACTACTCCACCGCTGAGGACGTAAGCAACCGTCCGAGTGACTTCGGTGCGTATCTGGATGCTCTGGAAGCCGAGGTTGCACGAGGCGGTCTTATTGTGTTCCACAACGGTcaca                                                                                                                       >  1:285635‑M2/59‑301 (MQ=255)`  
                                                                                                                                                                         `aCTCCACGCGGTGCAATCGTTGCCGATAAGACCAACATGATTCACACAGGTTTCTTGACAGTTGTTTCATATGAAGAGATTGTTAAGTCACGATAATC‑AATAGGAGAAATCAATATGATCGTTTCTGACATCGAAGCTAACGCCCTCTTAGAGAGCGTCACTAAGTTCCACTGCGGGGTTATCTACGACTACTCCACCGCTGAGTACGTAAGCTACCGTCCGAGTGACTTCGGTGCGTATCTGGATGCGCTGGAAGCCGAGGTTGCACGAGGCGGTCTTATTGTGTTCCACAACGGTcaca                                                                                                                       >  1:100278/1‑301 (MQ=255)`  
                                                                                                                                                                            `tccaacgaactggttgacagtgcatccactaaagttaccgaggctgcccgtgtggaaaaccaagctcaacagctttccaaattctttgagtaatcaaacaggagaaaccattatgATCGTTTCTGACATGGAAGCTAACGCCCTCTTAGAGAGCGTCACTAAGTTCCACTGCGGGGTTATCTACGACTACTCCACCGCTGAGTACGTAAGCTACCGTCCGAGTGACTTCGGTGCGTATCTGGATGCGCTGGAAGCCGAGGTTGCACGAGGCGGTCTTATTGTGTTCCACAACGGTCACAAg                                                                                                                     <  1:21050‑M2/186‑1 (MQ=255)`  
                                                                                                                                                                                 `cgaactggctgacagtgcatccactaaagttaccgaggctgcccgtgtggcaaaccaagctcaacagctttccaaattctttgagtaatcaaacaggagaaaccattatgATCGTTTCTGACATCGAAGCTAACGCCCTCTTAGAGAGCGTCACTAAGTTCCACTGCGGGGTTATCTACGACTACTCCACCGCTGAGTACGTAAGCTACCGTCCGAGTGACTTCGGTGCGTATCTGGATGCGCTGGAAGCCGAGGTTGCACGAGGCGGTATTATTGTGTTACACAACGGAAACAAGTATGa                                                                                                                <  1:265743‑M2/191‑1 (MQ=255)`  
                                                                                                                                                                                 `cagagtcaacagactggtccaacgacactgacttctgacaggtttcttgaCAGTTGTTTCATATGAAGAGATTGTTAAGTCACGATAATC‑AATAGGAGAAATCAATATGATCGTTTCTGACATCGAAGCTAACGCCCTCTTAGAGAGCGTCACTAAGTTCCACTGCGGGGTTATCTACGACTACTCCACCGCTGAGTACGTAAGCTACCGTCCGAGTGACTTCGGTGCGTCTCTGGTTGCGCTGGAAGCCGAGGTTGCACGAGGCGGTCTTATTGTGTTCCACAACGGTCACAAGTATGAc                                                                                                               >  1:422216‑M2/51‑301 (MQ=255)`  
                                                                                                                                                                                   `aactggctgacagtgcatccactaaagttaccgaggctgcccgtgtggcaaaccaagctcaacagctttccaaattctttgagtaatcaaacaggagaaaccattatgATCGTTTCTGACATCGAAGCTAACGCCCTCTTAGAGAGCGTCACTAAGTTCCACTGCGGGGTTATCTACGACTACTCCACCGCTGAGTACGTAAGCTACCGTCCGAGTGACTTCGGTGCGTATCTGGATGCGCTGGAAGCCGAGGTTGCACGAGGCGGTCTTATTGTGTTCCACAACGGTCACAAGTATGACg                                                                                                              <  1:15173‑M2/193‑1 (MQ=255)`  
                                                                                                                                                                                         `cgtacatggcatccactaaagttaccggggctgcccgtgtggcaaaccaagctcaacagctttccaaattctttgagtaatcaaacaggagaatctattatgATCGTTTCTGACATCGAAGCTAACGCCCTCTTAGAGAGCGTCACTAAGTTCCACTGCGGGGTTATCTACGACTACTCCACCGCTGAGTACGTAAGATACCGTCCGAGTGACTTCGGTGCGTATCTGGATGCGCTGGAAGCCGAGGTTGCACGAGGCGGTCTTATTGTGTTCCACAACGGTCACAAGTATGACGTTCCTg                                                                                                        <  1:281834‑M2/199‑1 (MQ=255)`  
                                                                                                                                                                                           `tgtgtgttccaattccacggtcgtctgtcaGGATTCTTGACAGTTGTTTCATATGAAGCTATTGTTTAGTCACGATAATT‑AATAGGGGAAATCAATATGATCGTTTCTGTCATCGCAGCTGCCGCCCTCGTAGGGGTCGTCCCTAAGTTCCACTGTGGGTTTATCTACGCCTACTCCACCGCTGAGTACGTAAGCTACCGTCCGAGTGACTTCGGTGCGTATCTGGCTTCGCTGGACGCCGAGGTTGTACTAGGCGGTATTATTGTGTTCCACAACGGTCACAAGTATGACGTTCCTGCAt                                                                                                     <  1:244847/273‑1 (MQ=255)`  
                                                                                                                                                                                            `acagtgcatccactaaagttaccgaggctgcccgtgtggcaaaccgagctcaacagctttccaaattctttgagtaatcaaacaggagaaaccattatgATCGTTTCTGACATCGAAGCTAACGCCCTCTTAGAGAGCGTCACTAAGTTCCACTGCGGGGTTATCTACGACTACTCCACCGCTGAGTACGTAAGCTACCGTCCGAGTGACTTCGGTGCGTATCTGGATGCGCTGGAAGCCGAGGTTGCACGAGGCGGTCTGATTGTGTTCCACAACGGTCACAAGTATGACGTTCCTGCAt                                                                                                     <  1:460312‑M2/202‑1 (MQ=255)`  
                                                                                                                                                                                            `aagagacagccactaaagttaccgaggctgcccgtgtggcaaaccaagctcaacagctttccaaattctttgagtaatcaaacaggagaaaccattatgATCGTTTCTGACATCGAAGCTAACGCCCTCTTAGAGAGCGTCACTAAGTTCCACTGCGGGGTTATCTACGACTACTCCACCGCTGAGTACGTAAGCTACCGTCCGAGTGACTTCGGTGAGTATCTGGATGCGCTGGAAGCCGAGGTTGCACGAGGCGGTCTTATTGTGTTCCACAACGGTCACAAGTATGACGTTCCTGCAt                                                                                                     <  1:407741‑M2/202‑1 (MQ=255)`  
                                                                                                                                                                                                `ggtccaacgacactgacttctgacaggattcttgaCAGTTGTTTCATATGAAGAGATTGTTAAGTCACGATAATC‑AATAGGAGAAATCAATATGATCGTTTATGACATCGAAGCTAACGCCCTCTTAGAGAGCGTCACTAAGTTCCACTGCGGGGTTATCTACGACTACTCCACCGCTGAGTACGTAAGCTACCGTCCGAGTGACTTCGGTGCGTATCTGGATGCGCTGGAAGCCGAGGTTGCACGAGGCGGTCTTCTTGTGTTCCACAACGGTCACAAGTATGAAGTTCCTGCATTGAcc                                                                                                >  1:115733‑M2/36‑301 (MQ=255)`  
                                                                                                                                                                                                         `aaaagttaccgaggctgcccgtgtggcaaaccaagctcaacagctttccaaattctttgagtaatcaaacatgagaaataaatatgATAGTTGCTGAAATCGAAGCTAACGCCCTCTTAGAGAGCTTCACGAAGTTCCACTGCGGGGTTATCTACGACTACTCCACCGCTGAGTACGTAAGCTACCGTCCTAGTGACTTCGGTGCGTATCTGGATGCGCTGGAAGCCGAGGTTGCACGAGGCGGTCTTATTGTGTTTCACAACGGTCACAAGTATGACGTTCCTGCATTGACCAAACTGGc                                                                                        <  1:505372‑M2/215‑1 (MQ=255)`  
                                                                                                                                                                                                             `gttaccgaggctgcccgtgtggcaaaccaagctcaacagctttccaaattctttgagtaatcaaacaggagaaaccattatgATCGTTTCTGACATCGAAACTAACGCCCTCTTAGAGAGCGTCACTAAGTTCCACTGCGGGGTTATCTACGACTACTACACCGCTGAGTACGTAAGCTACCGTCCGAGTGACTTCGGTGCGTATCTGGATGCGCTGGAAGACGAGGTTGCACGAGGCGGTCTTATTGTGTTCCACAACGGTCACAAGTATGACGTTCCTGCATTGACCAAACTggcactg                                                                                    >  1:323373‑M2/83‑298 (MQ=255)`  
                                                                                                                                                                                                                     `gacaggattcttgaCAGTTGTTTCATATGAAGAGATTGTTAAGTCACGATAATC‑AATAGGAGAAATCAATATGATCGTTTCTGACATCGAAGCTAACGCCCTCTTAGAGAGCGTCACTAAGTTCCACTGCGGGGTTATCTACGACTACTCTACCGCTGAGTACGTAAGCTACAGTCCGAGTGACTTCGGTGCGTATCTGGATGCGCTGGAAGCCGAGGTTGCACGCGGCGGTCTTATTGTGTTCCAAAACGGTCACAAGTATGACGTTCCTGCATTTACCAAACTGGCAAAGTTGCAATTg                                                                           >  1:467139‑M2/15‑301 (MQ=255)`  
                                                                                                                                                                                                                                    `aaaccaagctcaacagctttccaaattctttgagtaatcaaacaggagaaaccattatgATCGTTTCTGACATCGAAGCTAACGCCCTCTTAGAGAGCGTCACTAAGTTCCACTGCGGGGTTATCTCCGACTACTCCACCGCTGAGTACGTAAGCTACCGTCCGAGTGACTTCGGTGCGTATCTGGATGCGCTGGAAGCCGAGGTTGCACGCGGCGGTCTTATTGTGTTCCACCACGGTCACCAGTATGACGTTCCTGCATTGACCACACTGGCAAAGTTGCAATTGAACCGAAAGTtccc                                                             >  1:142884‑M2/60‑300 (MQ=255)`  
                                                                                                                                                                                                                                      `gacaggctcaacagctttccaaattctttgagtaatcaaacaggagaaaccattatgATCGTTTCTGACATCGAAGCTAACTCCCTCTTAGAGAGCGTCACTAAGTTCCACTGCGGGGTTATCTACGACTACTCCACCGCTGAGTACGTAAGCTACCGTCCGAGTGACTTCGGTGCGTATCTGGATGCGCTGGAAGCCGAGGTTGCACGAGGCGGTCTTATTGTGTTCCACAACGGTCACAAGTATGACGTTCCTGCATTGACCAAACTGGCAAAGTTGCAATTGAACCGAGAGTTCCAcc                                                           <  1:35864‑M2/244‑1 (MQ=255)`  
                                                                                                                                                                                                                                         `tttCATATGAAGAGATTGTTAAGTCACGATAATC‑AATAGGAGAAATCAATATGATCGTTTCTGACATCGAAGCTAACGCCCTCTTAGAGAGCGTCACTAAGTTCCACTGCGGGGTTATCTACGACTACTCCACCGCTGAGTACGTAAGCTACCGTCCGAGTGACTTCGGTGCGTATCTGGATGCGCTGGAAGCCGAGGTTGCACGAGGCGGTCTTATTGTGTTCCACAACGGTCACAAGTATGACGTTCCTGCATTGACCAAACTGGCAAAGTTGCAATTGAACCGAGAGTTCCACCTTcc                                                       <  1:367328/301‑1 (MQ=255)`  
                                                                                                                                                                                                                                           `gctcaacagctttccaaattctttgagtaatcaaacaggagaaaccattatgATCGTTTCTGACATCGAAGCTAACGCCCTCTTAGAGAGCGTCACTAAGTTCCACTGCGGGGTTATCTACGACTACTCCACCGCTGCGTACGTAAGCTACCGTCCGAGTGACTTCGGTGCGTATACGGGTGCGCTGGAAGCCGAGGTTGCACGAGGCGGGCTTATTGTGTTCCACAACGGTCACAAGTATGACGTTCCTGCATTGACCAAACTGGCAAAGttgcaattgaaccgagactgtctcttatac                                                      >  1:402704‑M2/53‑288 (MQ=255)`  
                                                                                                                                                                                                                                              `tatGAAGAGATTGTTAAGTCACGATAATCAAACAGGAGAAACCATTATGATCGTTTCTGACATCGAAGCTAACGCCCTCTTAGAGAGCGTCACTAAGTTCCACTGCGGGGTTATCTACGACTACTCCACCGATGAGTACGTAAGCTACCGTCCGAGTGACTTCGGTGCGTATCTGGATGCGCTGGAAGCCGAGGTTGCACGAGGCGGTCTTATTGTGTTCCACAACGGTCACAAGTATGACGTTCCTGCATTGACCAAACTGGCAAAGTTGCAATTGAACCGAGAGTTCCACCTTCCTCGt                                                   >  1:45559/1‑301 (MQ=255)`  
                                                                                                                                                                                                                                                    `ctttccaaattctttgagtaatcaaccaggagaaaccattatgATCGTTTCTGACATCGAAGCTAACTCCCTCTTAGAGAGCGTCACTAAGTTCCACTGCGGGGTTATCTACGACTACTCCACCGCTGAGTACGTAAGCTACCGTCCGAGTGACTTCGGTGCGTATCTGGATGCGCTGGAAGCCGAGGTTGCACGAGGCGGTCTTATTGTGTTCCACAACGGTCACAAGTATGACGTTCCTGCATTGACCAAACTGGCAAAGTTGCAATTGAACCGAGAGTTCCACCTTCCTCGTGAGAAc                                             <  1:375922‑M2/258‑1 (MQ=255)`  
                                                                                                                                                                                                                                                    `cttgccaaatgctttaaggaatcaaacaggagaaaccattatgAGCGTTTCTGACATCGAAGCTAACGCCATCTTAGAGAGCGTCACTAAGTTCCACTGCGGGGTTATCTACGACTACTCCACCGCTGAGTACGTAAGCTACCGTCCGAGTGACTTAGGTGCGTATCGGGATGCGCTGGAAGCCGAGGTTGAACGAGGCGGTCTTATTGTGTTCCACAACGGTCACAAGTATGACGTGCCTGCATTGACCAAACTGGCAAAGTGGCAATTGAACCGAGAGTTCCACCTTCCTCGTGAGAAc                                             <  1:254238‑M2/258‑1 (MQ=255)`  
                                                                                                                                                                                                                                                            `tAAGTCACGATAATC‑AATAGGAGAAATCAATATGATCGTTTCTGACATCGAAGCTAACGCCCTCTTAGAGAGCGTCACTAATTTCCACTGCGGGGTTATCTACGACTACTCCACCGCTGATTACGTAAGCTACCGTCCGAGTGACTTCGGTGCGTATCTGGATGCGCTGGAAGCCGAGGTTGCACGAGGCGGTCTTATTGTGTTCCACAACGGTCACAAGTATGACGTTCCTGCATTGACCAAACTGGCAAAGTTGCAATTGAACCGAGAGTTCCACCTTCCTCGTGAGAACTGTATTGac                                    <  1:231062/301‑1 (MQ=255)`  
                                                                                                                                                                                                                                                             `ttctttgagtaatcaaacaggagaaatcaatatgATCGTTTCTGACATCGAAGCTAACGCCCTCTTAGAGAGCGTCACTAAGTTCCACTGCGGGGTTATCTACGACTACTCCACCACTGAGTACGTAAGCTACCGTCCGAGTGACTTCGGTGAGTATCTGGATGCGCTGGAAGCCGAGGTTGCACGAGGCGGTCTTATTGTGTTCCACAACGGTCACAAGTATGACGTTCCTGCATTGACCAAACTGGCAAAGTTGCAATTGAACCGCGAGTTCCACCTTCCTCGTGAGAACTGTATTGac                                    <  1:440006‑M2/267‑1 (MQ=255)`  
                                                                                                                                                                                                                                                              `aGTCACGATAATC‑AATAGGAGAAATCAATATGATCGTTTCTGACATCGAAGCTAACGCCCTCTTAGAGAGCGTCACTAAGTTCCACTGCGGGGTTATCTACGACTACTCCACCGCTGAGTACGTAAGCTACCGTCCGAGTGACTTCGGTGCGTATCTGGATGCGCTGGAAGCCGAGGTTGCACGAGGCGGTCTTATTGTGTTCCACAACGGTCACAAGTATGACGTTCCTGCATTGACCAAACTGGCAAAGTTGCAATTGAACCGAGAGTTCCACCTTCCTCGTGAGAACTGTAtctgtct                                  >  1:188426/1‑295 (MQ=255)`  
                                                                                                                                                                                                                                                               `gtaaCGATAATC‑AATAGGAGAAATCAATATGATCGTTTCTGACATCGAAGCTAACGCCCTCTTAGAGAGCGTCACTAAGTTCCACTGCGGGGTTATCTACGACTACTCCACAGCTGAGTACGTAAGCTACCGTCCGAGTGACTTCGGTGCGTATCTGGATGCGCTGGAAGCCGAGGTTGCACGAGGCGGTCTTATTGTGTTCCACAACAGTCACAAGTATGACGTTCCTGCATTGACCAAACTGGCAAAGTTGCAATTGAACCGAGAGTTCCACCTTCCTCGTGAGAACTGTATTGACAcc                                 <  1:130023/298‑1 (MQ=255)`  
                                                                                                                                                                                                                                                                     `gtAATC‑AATAGTAGAAATCAATATGATCGTTTCTGACATCGAAGCTTACTCCCTCTTAGAGAGCGTCACTAAGTTCCACTTCGTGTTTATCTACGACTACTCCACCCCTGAGTACGTAAGCTACCGTCCGAGTGACTTCGGTTCGTATCTGGATGCGCTGGAAGCCGATGTTGCACGAGGCGGTCTTATTGTGTTCCTCAACGGTCACAAGTATGACGTTCCGGCCTTGACCAAACTGTCAAAGTTGCAATTTAACTGCAAGTTCAACCTTCCTCGTGCGCACTGTATTCACTCCCtgtct                           >  1:501050/2‑297 (MQ=255)`  
                                                                                                                                                                                                                                                                      `tgtgtataagagacagaccattatgCTCGGTTCTGACATCGAAGCTAACGCCCTCTTAGAGAGCGTCACTAAGTTCCACTGCGGGGTTATCTACGACTACTCCACCGCTGAGTACGTAAGCTACCGTCCGAGTGACTTCGGTGCGTATCTGGATGCGCTGGAAGCCGAGGTTGCACGAGGCGGTCTTATTGTGTTCCACAACGGTCACAAGTATGACGTTCCTGCATTGACCAAACTGGCAAAGTTGCAATTGAACCGAGAGTTCCACCTTCCTCGTGAGAACTGTATTGACACCCTtgtg                           <  1:14622‑M2/276‑1 (MQ=255)`  
                                                                                                                                                                                                                                                                       `aatcaaacaggagaaatcaataggATCGTTTCTGACATTGAAGCTAACGCCCTCTTAGAGAGCGTCATTAAGTTCCACTGCGGGGTTATCTACGACTACGCCACCGCTGAGTACGTAAGCTACCGTCCGAGTGACTTCGGTGCGTATCTGGATGCGCTGGAAGCCGAGGTTGCACGATGCGGTCTTATTGTGTTCCACAACGGTCACAAGTATGACGTTCCTGCATTGACCAAACTGGCAAAGTTGCAATTGAACCGAGAGTTCCACCTTCCTCGTGAGAACTGTATTGACACCCTTgtgt                          <  1:106238‑M2/277‑1 (MQ=255)`  
                                                                                                                                                                                                                                                                       `aaacaaaccagacaaaTCAATATGTGCGTTTCTGAACTCGAAACTTACGCCCTCTTAAGGAGCGTCAATAATTTCCACTGCGGTGTTATCTACGACTAATCCACCGCTGAGTACGTAAGCTACCGTCCGAGTGACTTCGGTGCGTATCTGGATGCGCTGGAAGCCGAGGTTGCACGAAGCGGTCTTATTGTGTTCCACAACGGTCACAAGTATGACGTTCCTGCATTGACCAAACTGGCAAAGTTGCAATTGAACCGAGAGTTCCACCTTCCTCGTGAGAACTGTATTGACACCCTTgtgt                          <  1:329036/288‑1 (MQ=255)`  
                                                                                                                                                                                                                                                                        `aacaagcaggaacaatatatatGATAGTTTCTGACATGGAAGCTAACGCCCTC‑GAGAGAGCGTCCCTAAGTTCCATGGCGTGCTTATCTAGGTCTCCTCCACCGCAGAGTACGTGAGGGAGCGTCCGAGTGACTTCGGGGTGTGTCTGGATGCGCTGGAAGCCGAGGTTGCACGAGGCGGTCTTATTGTGTTCCACAACGGTTACAAGTATGAAGTTGCTGCATTGACCAAACTGGCAAAGTTGCAATTGACCCGAGAGTTCCACCTTCCTCATGAGAACTGTATTGACACCCTTGtgttg                        <  1:109200/283‑1 (MQ=255)`  
                                                                                                                                                                                                                                                                                `ggagaaaccattatgATCGTTTCTGACATCGAAGCTAACGCCCTCTTAGAGAGCGTCACTAAGTTCCACTGCGGGGTTATCTACGACTACTCCACCGCTGAGTACGTAAGCTACCGTCCGAGTGACTTCGGTGAGTATCTGGATGCGCTGGAAGCCGAGGTTGCACGAGGCGGTCTTATTGTGTTCCACAACTGTCACAAGTATGACGTTCCTGCATTGACCAAACTGGCAAAGTTGCACTTGAACCGAGAGTTCCCCCTTCCTCGTGAGCACTGTCTTGACACCCTTGTGTTGtcactgt                 >  1:43049‑M2/16‑298 (MQ=255)`  
                                                                                                                                                                                                                                                                                `ggAGAAATCAATATGATCGTTTCTGACATCGAAGCTAACGCCCTCTTAGAGAGCGTCACTAAGTTCCACTGCGGGGTTATCTTCGACTACTCCACCGCTTAGTACGTAAGCTACCGTCCGAGTGACTTCGGTGCGTATCTGGATGCGCTGGAAGCCGAGGTTGCACGAGGCGGCCTTATTGTGTTCCACAACGGTCATAAGTATGACGTTCCTGCATTGACCTAACTGGCTAAGTTGCAATTGAACCGAAAGTTCCACCTTCCTCTTGACAACTGTATTGACACCCTTATGTTGTCACGtt                 >  1:412000/1‑301 (MQ=255)`  
                                                                                                                                                                                                                                                                                `ggAGAAATCAATATGATCGTTTCTGACATCGAAGCTAACGCCCTCTTAGAGAGCGTCACTAAGTTCCACTGCGGGGTTATCTACGACTACTCCACCGCTGAGTACGTAAGCTACCGTCCGAGTGACTTCGGTGCGTATCTGGATGCGCTGGAAGCCGAGGTTGCACGAGGCGGTCTTATTGTGTTCCACAACGGTCACAAGTATGACGTTCCTGCATTGACCAACCTGGCAAAGTTGCAATTGAACCGAGAGTTCCACCTTCCTCGTGAGAACTGTATTGACACCCTTGTGTTGTCACGtt                 >  1:138663/1‑301 (MQ=255)`  
                                                                                                                                                                                                                                                                                     `aaTCAATATGATCGTTTCTGACATCGAAGCTAACGCCCTCTTAGAGTGCGTCACTAAGTTCCACTGCGGGGTTATCTACTACTACTCCACCGCTGAGTACGTAAGCTACCGTCCTAGTGACTTCGGTGCGTATCTGGATGCGCTGGAAGCCGAGGTTGCACGAGGCGGTCTTATTGTGTTCCACAACGGTCACAAGTATGACGTTCCTGCATTGACCAAACTGGCAAAGTTGCAATTGAACCGAGAGTTCCACCTTCCTCGTGAGAACTGTATTGACACCCTTGTGTTGTCACGTTTGatt            <  1:261153/301‑1 (MQ=255)`  
                                                                                                                                                                                                                                                                                       `ccattatgATCGTTTCTGACATCGAAGCTAACGCCCTCTTAGAGAGCGTCACTAAGTTCCACTGCGGGGTTATCTACGACTACTCCACCGCTGAGTACGTAAGCTACCGTCCGAGTGACTTCGGTGCGTATCTGGATGCGCTGGAAGCCGAGGTTGCACGAGGCGGTCTTATTGTGTTCCACAACGGTCACAAGTATGACGTTCCTGCATTGACCAAACTGGCAAAGTTGCAATTGAACCGAGAGTTCCACCTTCCTCGTGAGAACTGTATTGACACCCTTGTTTTGTCACGTTTGattca          >  1:206865‑M2/9‑301 (MQ=255)`  
                                                                                                                                                                                                                                                                                          `ttatgATCGTTTCTGACATCGAAGCTAACGCCCTCTTAGAGAGCGTCACTAAGTTCCACTGCGGGGTTATCTACGACTAATCCACCGCTGAGTACGTAAGATACCGTCCGAGTGACTTCGGTGAGTATCTGGATGCGCTGGAAGCCGAGGTTGCACGAGGCGGTCTTATTGTGTTCCACAACGGTCACAAGTATGACGTTCCTGCATTGACCAAACTGGCAAAGTTGCAATTGAACCGAGAGTTCCACCTTCCTCGTGAGAACTGTATTGACACCCTTGTGTTGTCCCgtttgattacttc       >  1:277725‑M2/6‑296 (MQ=255)`  

                                                                                                                                                                                                                                                                                               `|`                                                                                                                                                                                                                                                                                                              
`GAAGAAACCAGAAGTTACCAAGCTGAGTCATAAGGTGTTCGATAAGGACACTATGACCCACATCAAGGCTGGTGATTGGGTTAAGGTTGACATGGGAGTTGTTGGTGGATACGGCTACGTCCGCTCAGTTAGTGGCAAATATGCACAAGTGTCATACATCACAGGTGTTACTCCACGCGGTGCAATCGTTGCCGATAAGACCAACATGATTCACACAGGTTTCTTGACAGTTGTTTCATATGAAGAGATTGTTAAGTCACGATAATC‑AATAGGAGAAATCAATATGATCGTTTCTGACATCGAAGCTAACGCCCTCTTAGAGAGCGTCACTAAGTTCCACTGCGGGGTTATCTACGACTACTCCACCGCTGAGTACGTAAGCTACCGTCCGAGTGACTTCGGTGCGTATCTGGATGCGCTGGAAGCCGAGGTTGCACGAGGCGGTCTTATTGTGTTCCACAACGGTCACAAGTATGACGTTCCTGCATTGACCAAACTGGCAAAGTTGCAATTGAACCGAGAGTTCCACCTTCCTCGTGAGAACTGTATTGACACCCTTGTGTTGTCACGTTTGATTCATTCCAACC  >  NC_001604.1/14070‑14656`

  

Alignment Legend

Aligned base mismatch/match (shaded by quality score): `ATCG/ATCG` `< 3 ≤` `ATCG/ATCG` `< 9 ≤` `ATCG/ATCG` `< 18 ≤` `ATCG/ATCG` `< 33 ≤` `ATCG/ATCG` `< 38 ≤` `ATCG/ATCG`

Unaligned base: `atcg`    Masked matching base: `atcg`    Alignment gap: `‑`    Deleted base: `‑`

Reads not counted as support for junction

read\_name Not counted due to insufficient overlap past the breakpoint.

read\_name Not counted due to not crossing MOB target site duplication.
